# Female Black‐Capped Chickadees (*Poecile atricapillus*) Expand Foraging Both Temporally and Spatially to Mitigate Interference Competition

**DOI:** 10.1002/ece3.73972

**Published:** 2026-07-10

**Authors:** Elène Haave‐Audet, Kimberley J. Mathot

**Affiliations:** ^1^ Department of Biological Sciences University of Alberta Edmonton Alberta Canada; ^2^ Département des Sciences Biologiques Université du Québec à Montréal Montreal Quebec Canada

**Keywords:** Aves, caching, dominance, Paridae, pulsed resources, uncertainty management

## Abstract

Group living provides many potential benefits but can also incur important costs via competition for access to resources. Even in the absence of resource depletion, interference competition can reduce access to resources, and interference competition is not expected to affect all group members equally. In groups with dominance hierarchies, dominant individuals have priority access to resources and are therefore expected to experience lower costs of interference competition compared to subordinate individuals. Here, we assessed the effect of manipulating the number of feeders available with ad libitum food, which we assumed would affect competition intensity, on total daily feeder visits in a marked population of 126 black‐capped chickadees ( *Poecile atricapillus* ). As predicted, we found strong evidence of interference competition: reducing feeder availability by 50% reduced average per capita daily visits to feeders in our population by circa 50%. Because chickadee flocks exhibit linear dominance hierarchies, with males being dominant over females, and at least in some populations, adults being dominant over immatures, we also investigated whether there was evidence for sex‐ and/or age‐related differences in the strength of interference competition. We found that reductions in total daily feeder visits under lower manipulated food availability were similar for all age‐sex categories, suggesting that females and immatures did not experience higher interference competition despite their subordinate status relative to males and adults. Post hoc analyses revealed that adult females expanded their foraging area, while immature females increased both the length of the foraging window and foraging area when the availability of feeders was reduced. We suggest that these results may indicate that females invest more to mitigate competition and manage unpredictable access to food due to their subordinate status.

## Graphical abstract

Reducing feeder availability reduces per capita intake in black‐capped chickadees, presumably via interference competition. We show that chickadees mitigate this increased competition by increasing the time spent foraging and increasing space use. However, these effects are greatest for subordinates (females and/or immatures); immature females had significantly greater increases in total time spent foraging compared to other age‐sex classes, while both adult and immature females increased foraging area significantly more compared to adult males, with immature males showing intermediate increases in space use.

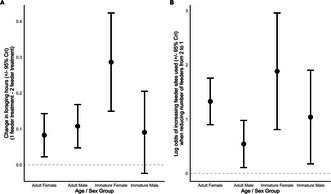

## 1 Introduction

Animals can gain a variety of benefits from living in groups, including increased vigilance against predators (Lima 1995; Lima and Dill 1990; Pulliam 1973), more efficient search tactics to find resources (Giraldeau and Caraco 2000), and increased access to mates (Danchin and Wagner 1997). However, living in groups can also incur costs, including increased attack rates by predators when larger groups are more conspicuous, increased parasite burden, and increased competition for food (Krause and Ruxton 2002). Increased competition for food is generally considered the most significant cost associated with group living, and the bigger the group, the faster resources may become depleted (Krause and Ruxton 2002).

However, even when resources are not depleted, group living can lead to reduced intake rate via interference competition, whereby some individuals restrict others' access to food (Goss‐Custard 1980). Theory predicts that both group size and resource availability (i.e., the quantity and/or quality of resources) influence the degree of interference competition in groups of foragers (Stillman, Goss‐Custard, and Caldow 1997; Ydenberg et al. 1986), and several empirical studies in birds have found support for these predictions (Bijleveld et al. 2012; Cresswell 1998; Dolman 1995; Kotrschal et al. 1993; Maziarz et al. 2023; Stillman, Goss‐Custard, Clarke, and Durell 1997). For example, Bijleveld et al. (2012) evaluated evidence for interference competition in red knots ( *Calidris canutus islandica* ). They found that despite ensuring negligible patch depletion via their experimental design, intake rate and search efficiency both decreased with increasing group size, and these effects were stronger when food density was lower (Bijleveld et al. 2012).

Interference competition is not expected to affect all group members equally; socially dominant individuals have priority access to food and are able to exclude subordinate group members (Drews 1993; Rowell 1974). Thus, the strength of interference competition is expected to be greater for subordinates compared to dominants. Greater interference competition for subordinates can occur via overt aggression by dominant individuals towards subordinates (Stillman, Goss‐Custard, and Caldow 1997), but can also occur if subordinates avoid interactions with dominants to mitigate the costs of aggression (Bijleveld et al. 2012; Gyimesi et al. 2010; Stillman, Goss‐Custard, and Caldow 1997; Vahl and Kingma 2007). In many animals, energy requirements can be strongly shaped by environmental conditions. For example, low temperatures (below thermoneutral) and high wind speeds both increase energy demand by increasing the cost of thermoregulation (Wolf and Walsberg 1996). Thus, for a given group size and given food abundance, colder and/or windier conditions may increase the strength of interference competition.

In our study, we examined the effects of intraspecific competition on foraging rates in a free‐living population of black‐capped chickadees ( *Poecile atricapillus* ). Black‐capped chickadees (hereafter chickadees) form stable winter flocks and exhibit stable linear dominance hierarchies, with males being dominant over females (Smith 1992). In some populations, adult chickadees are dominant over immatures (Desrochers 1989; Desrochers et al. 1988), though some studies report age effects in males only (Ramsay and Ratcliffe 2003; Schubert et al. 2007; Smith 1976), or no age‐related effects at all (Brittingham and Temple 1992; Wilson 2001). Dominant chickadees will displace subordinate individuals at a foraging patch, while subordinate individuals wait for dominant birds to leave the patch before exploiting it (Ficken et al. 1990). Chickadees readily visit artificial feeders on their foraging territory, making them amenable to studies where food availability is manipulated. Throughout their range across the northern United States and Canada, chickadees consistently experience temperatures below their thermoneutral zone during winter, and they experience large variation in environmentally imposed energetic costs at this time of year.

We investigated the effect of changing the intensity of competition on chickadees by manipulating food availability at the study site. We alternated between providing non‐depleting black‐oil sunflower seeds at two feeders versus one feeder at eight feeder sites within the study area. If interference competition was lower when two feeders were provided per site compared to a single feeder being provided per site, we predicted that per capita intake rates would decline when only one feeder was provided. Because our experiments were conducted over the winter when chickadees experience large fluctuations in energy requirements due to changing ambient temperatures, we also investigated temperature‐related changes in foraging rates within each feeder treatment. Overall, we predicted that daily foraging would be negatively related to ambient temperature, as lower temperatures necessitate higher intake to meet energy expenditure (Bonter et al. 2013; Hawkshaw et al. 2025; Kessel 1976; LaRocque et al. 2024; Latimer et al. 2018), but that within the two‐feeder treatment (i.e., under low competition), chickadees would have greater scope to increase foraging rate with increasing energetic requirements relative to the single‐feeder (i.e., high competition) treatment (i.e., we predicted a stronger effect of temperature under low competition). Additionally, we tested whether there was support for sex‐related differences in the response to competition, based on the fact that chickadees have strong sex‐related differences in dominance (Desrochers 1989; Smith 1976). We also investigated age effects, because at least in some chickadee populations, adults are dominant over immatures (Desrochers 1989; Desrochers et al. 1988). If the strength of interference competition is mediated by dominance, we predicted that males and/or adults would exhibit smaller decreases in foraging rate as food availability decreased compared with females and/or immatures.

## 2 Methods

### 2.1 Study Site and Population

We studied a free‐living population of black‐capped chickadees at the University of Alberta Botanic Garden, near Devon, Alberta, Canada (53°24′27″ N, 113° 45′ 41″ W). The University of Alberta Botanic Garden is a 0.97 km^2^ property that consists of several small cultivated gardens (0.32 km^2^), and areas of mixed forest (0.65 km^2^). The chickadee work is carried out in the mixed forest areas of the property where aspen ( *Populus tremuloides* ) and Scots pine ( *Pinus sylvestris* ) are the dominant tree species. Chickadees at the study site were individually marked using passive‐integrative transponder (PIT) tags affixed to a leg band on the tarsus (detailed description below). Foraging behaviour was measured using a set of experimental feeders equipped with radio‐frequency identification (RFID) antennae and readers, which detected individual PIT tags when the birds visited the feeders (see Arteaga‐Torres et al. 2020 for a detailed description). Feeders were protected with chicken wire (see Figure S1), which prevented squirrels from accessing feeders, as well as non‐focal bird species. The only other species present in the study area in winter which is small enough to use the feeders alongside chickadees is the red‐breasted nuthatch ( *Sitta canadensis* ). However, red‐breasted nuthatches are present at low numbers, with an estimated population of between 4 and 10 individuals in non‐eruptive years, such as the present study year (J.J. Wijmenga, personal communication), and thus do not create important competition for feeders.

We studied interference competition at eight feeder sites. Feeder sites were at least 270 m apart throughout the study area, to approximate chickadee non‐breeding flock territory size (Smith 1992). Previous work has shown that most individuals use either a single feeder site, or exhibit a strong preference for a single site, consistent with the assumption that feeder sites represent unique winter territories (LaRocque et al. 2024). Individuals were captured and marked at the feeder sites using mist‐nets from September to December 2017, March, November and December 2018, and March and November 2019. At initial capture, birds were marked with United States Geological Survey (USGS) metal bands. As part of standard data collection for this population, we collected a blood sample from the brachial vein to molecularly sex individuals, because chickadees are sexually monomorphic (Smith 1992). We also affixed a leg band embedded with EM4102 PIT tags on newly captured individuals (8 mm × 2 mm, Eccel Technology Ltd., UK), along with a second colour band in a unique combination to visually identify individuals. During catching, we estimated age based on plumage characteristics (Pyle 1997). Birds assigned the age ‘first calendar year’ during catching in Fall 2019 are referred to as ‘immature’ for this study, while birds assigned the age ‘>1st calendar year’ in Fall 2019 and birds that were captured prior to Fall 2019 are referred to as ‘adult’.

### 2.2 Experimental Design and Data Selection

We studied the effects of interference competition on foraging behaviour between 29 November 2019 and 4 March 2020. The experiments did not start until three weeks after the annual catching effort was complete, to avoid carry‐over effects from catching (see Macleod and Gosler 2006). During the post‐catching/pre‐experiment period, we first provided a constant source of black‐oil sunflower seeds at all eight feeder sites for two weeks. After this 2‐week period, we installed a second feeder at each of the eight feeder sites, approximately 10–15 m from the first feeder, to ensure that the second feeder was also on the foraging territory of a given flock and that birds would be able to find and exploit the new feeder. Both feeders remained empty for one week prior to the start of the experiment.

During the experimental period, we manipulated food availability at each site by having either both feeders full, or a single feeder full, which we expected would affect the intensity of interference competition at the feeder site. By manipulating whether both feeders or only one of the two feeders was full at a given time, we implemented treatments of two ad libitum feeders per site (which we assumed created low competition) and one ad libitum feeder per site (high competition). The same treatment was in effect at all feeder sites at any given time. The two‐feeder (i.e., low competition) treatment was in effect for four consecutive days, at four different periods during the study (see Table S1 for a detailed timeline). The single‐feeder (i.e., high competition) treatment was also in effect for four days per feeder per site; however, this treatment was repeated eight times instead of four since we switched which of the two feeders was full after the first four days as part of another study focused on understanding sampling behaviour at feeders while empty (Haave‐Audet et al. 2024). However, for the current study, we only retained the first four days of the single‐feeder treatment for analysis to ensure consistency in the length of the two treatments. Each successive pair of two‐feeder treatment and single‐feeder treatment within a site is called a ‘trial’, with four trials conducted at each feeder site synchronously over the course of the study. Between each trial, we left both feeders empty for 12 days at each site, to avoid the inflating effects on survival of a continuous food source at the study site (Robb et al. 2008; Wilson 2001).

Our study design was such that treatment order was the same for each of the four trials, with two‐feeder treatments occurring first, followed by the single‐feeder treatment. Having a fixed treatment order has both advantages and disadvantages. The major advantage of this approach was that it meant that we were able have a standard reference level to determine whether birds should be included in each trial. If birds were present with two feeders per site (i.e., low competition), they had discovered that the feeders were full, and subsequent absence during one feeder per site (i.e., high competition) could be assumed to indicate competitive exclusion. Birds that were absent during the two‐feeder per site treatment but appeared during the single‐feeder per site treatment could reasonably be assumed to have not discovered the feeders during the two‐feeder per site period, and therefore, could not be included in the single‐feeder per site period as we could not calculate the change in feeding rate due exclusively to the change in the assumed manipulated competition level. In contrast, if our experimental design had allowed for the single‐feeder per site periods to occur first, absence of individuals during the single‐feeder treatment could indicate either competitive exclusion or failure to discover the feeders, making it difficult to isolate the effect of manipulations of competition level on feeding rates. For this reason, it would also not have been appropriate to randomise treatment order and subsequently restrict analyses to birds that were present during both treatments, as this might disproportionately remove birds that were strongly affected by higher competition, and hence absent during the single‐feeder treatment. However, one potential disadvantage of the fixed treatment order employed here is that because the two‐feeder treatment was always preceded by 12 days with no food supplementation, chickadees might exhibit higher feeding rates upon rediscovery of feeders that were unrelated to the treatment per se. For example, high feeder visit rates upon feeder rediscovery could occur if chickadees replenish caches following periods without supplemental food. This would lead to higher feeder visit rates during the two‐feeders per site period that might not reflect treatment‐related differences in feeding rate, but rather continuous temporal trends in caching following rediscovery of a reliable food source. Therefore, we modelled days since treatment start to evaluate whether treatment effects were better explained by continuous temporal effects (see Section 2.3).

During both treatments, we measured foraging rate by counting the total daily number of visits to full feeders across all sites, per individual, per day. For the two‐feeder treatment, we included visits to both full feeders at all sites in an individual's daily total, while during the single‐feeder treatment, we only included visits to the single full feeder at each site and excluded visits to the empty feeder as these visits did not provide a measure of food acquisition. Based on video recordings at feeders used to validate the RFID data, we determined that chickadees take a single sunflower seed per visit, with less than 1% of visits involving a chickadee leaving without taking a seed (e.g., due to displacement; Ipshita Gayen, unpublished data). Thus, in the present study, count of visits is expected to be strongly correlated with the count of sunflower seeds taken. Furthermore, previous work in chickadees using similar feeder designs to those used in the current study found that the minimum time taken by an individual chickadee to make two unique visits to a feeder is 12 s (Arteaga‐Torres et al. 2020). Thus, successive RFID detections of the same individual that occurred within 12 s of each other were consolidated into a single visit. On days where an individual was not detected, we determined whether the individual was detected at a full feeder at any earlier point during that trial (including both treatments), and if it was, we assigned zero daily visits for the day that the bird was not detected at feeders. If the individual was only detected partway through a trial, daily feeder visits for all days preceding the first detection within a trial (including both treatments) were scored as ‘NA’. We did not assign visit rates of ‘0’ to days in a trial prior to the first feeder visit within that trial, because we could not know whether the lack of feeder visits was because birds had not detected that the feeders were full, or a decision to not use feeders that had been rediscovered as full. Similarly, birds that were absent from feeders for an entire trial were not assigned ‘0’ daily feeder visits, but were scored as ‘NA’. We excluded feeder visit data on the day each treatment was initiated (zero days since start of treatment) because treatment changes occurred partway through the foraging period and, therefore, we did not have total daily feeder visits for each treatment on those days (see Table S1). RFID batteries and SD cards were changed at the same time as feeders were (re‐)filled or emptied during treatment changes. The feeders were able to hold sufficient sunflower seeds that feeders never became depleted between these changes.

Capture methods and food experiment manipulations were conducted in accordance with the Canadian Council on Animal Care guidelines and were approved by the University of Alberta Animal Care and Use Committee (AUP00002210). Bird banding was approved by the Bird Banding Office in Canada (permits 10,936 and 10936A), and field work was approved by Alberta Fish and Wildlife Capture Research (17658720).

### 2.3 Analyses

All analyses were conducted in R (v 4.5.2) using RStudio (v 2026.1.1.403, R Core Team 2025). We explored sources of variation in the count of daily feeder visits using linear mixed effects models using the *lmer* function from the package ‘lme4’ (Bates et al. 2015) with Gaussian error distributions. We included the following fixed effects: feeder treatment (two levels: two feeders per site or one feeder per site), ‘age‐sex’ (four levels: adult female, adult male, immature female, immature male), days since treatment start (continuous), mean daily ambient temperature (continuous), the interaction between feeder treatment (two vs. one) and age‐sex, the three‐way interaction between feeder treatment, days since the start of the treatment, and age‐sex, and the three‐way interaction between feeder treatment, average daily temperature, and age‐sex. Average daily temperature data were extracted from the Environment and Climate Change Canada Historical Data Website ([https://climate.weather.gc.ca/index_e.html] accessed on 2021‐02‐09 from the Edmonton International Airport weather station). Prior to analyses, temperature was centred and standardised using the *scale* function in R, which centres temperature around the average value in the data set and divides by one standard deviation. As such, intercept estimates are for average daily temperatures in the dataset (i.e., −11.53°C), and the effect size for temperature is the change in response variable per one standard deviation change (i.e., 6.72°C) in temperature. Days since start (range 1–3) was left‐zeroed prior to analysis by subtracting 1 from each value, so that model intercepts were estimated on Day 1. We included individual ID and trial number as random effects in the models to account for the effect of repeated measures at each of these levels on the variance structure of the data. Trial number (four levels) refers to each paired treatment (two ad libitum feeders per site followed by one ad libitum feeder per site). Daily feeder visit data were Poisson distributed (see Figure S2). Therefore, we verified that the model satisfied assumptions of normally distributed residuals (based on visual inspection of a histogram of model residuals), which it did. Although the Gaussian model was heteroscedastic (based on visual inspection of plots of fitted versus predicted values), previous simulation studies have shown that estimates from linear mixed effects models are generally robust to violations of model assumptions (Schielzeth et al. 2020). Nonetheless, we verified that model inferences were the same when modelled with a Poisson error distribution, which they were (see Text S1 and Table S2). Estimates derived from linear mixed effects models have the advantage of being more intuitive to interpret, because they are on the observed scale, therefore, we present results from the linear model using untransformed daily feeder visits in the main text.

We used the *sim* function from the ‘arm’ package to simulate the posterior distribution of the model parameters from 1000 simulations (Gelman and Hill 2006; Gelman and Su 2021). The *sim* function uses a default flat, non‐informative prior (Gelman and Hill 2006; Gelman et al. 2008). We interpreted estimates with 95% credible intervals that exclude zero as providing strong support for an effect. Estimates centred on zero were interpreted as providing no support for an effect, or strong support for lack of an effect. For estimates that were biased away from zero, but whose 95% CrI overlapped zero, we calculated the proportion of overlap (pr) to allow us to infer the level of support for interpretation of an effect. As an example, pr = 0.15 corresponds to five times greater support for the interpretation of an effect in the reported direction compared to an effect in the opposite direction (i.e., 0.85/0.15 = 5.7) (Marsman and Wagenmakers 2017). Non‐overlapping 95% CrIs from two independent groups (such as adult males and adult females) were interpreted as providing strong support that the effects were different between the two groups when the 95% CrIs did not overlap. Arms of 95% CrI that have a proportion overlap of up to 0.58 corresponds to a one‐tailed *p*‐value of 0.047 (see Cumming and Finch 2005). Thus, for cases where 95% CrIs of independent groups overlapped, we calculated the difference between estimated effects and the 95% CrI of the difference and interpreted the estimated difference as described above; 95% CrI not overlapping zero were interpreted as providing strong support that the effects were different, and estimated differences whose 95% CrI overlapped zero by up to pr = 0.15 were interpreted as providing moderate support that the effects were different.

Because the lack of sex‐ and/or age‐related differences in response to treatment were unexpected based on sex‐ and age‐related differences in dominance in chickadees (see Section 3), we conducted post hoc analyses to evaluate whether females and/or immatures adjusted other aspects of their behaviour to allow them to achieve similar daily feeder visits as males and/or adults under reduced food availability, despite being the subordinate sex and/or age classes. Specifically, subordinates are expected to have longer foraging periods (Krams 2000), to forage at risker times (Alanärä et al. 2002; Lahti et al. 1997), and/or forage in riskier places (Barluenga et al. 2008; Ekman 1987; Koivula et al. 1994; Schneider 1984; Slotow and Paxinos 1997) to help them achieve their required intake rates. Therefore, we evaluated whether female and/or immature birds increased the length of their daily foraging period, or the number of feeder sites used, relative to males and/or adults to mitigate potential competition. We predicted that females and immatures would have a longer daily foraging period than males and adults and/or use a larger number of feeder sites, particularly in the single‐feeder treatment. To test these predictions, we constructed two separate mixed effects models. First, we constructed a linear mixed effect model with the daily number of foraging hours as the response variable and a Gaussian error distribution. To get an estimate of the length of the foraging window, we calculated the number of hours between the first and last observation of the day, per individual. Foraging hours closely matches hours of daylight, though some birds had notably short foraging windows, suggesting that the feeders were not their primary food source (see Figure S3). Thus, we restricted our analyses to birds that had foraging hours within ±2.5 h of daylength, which removed *n* = 186 observations from the overall dataset of *n* = 2340 observations (i.e., 7.9%). We acknowledge that this filtering decision was subjective, and verified that results were not unduly influenced by this decision, which they were not (see Table S3). However, model fit was better with the filtered data, and therefore, we present results using the filtered data in the main text. We additionally verified that filtering the data did not change the interpretations of the main effect of feeder treatment on total daily feeder visits, which it did not (see Table S4). Second, we modelled daily feeder sites visited as the response variable using the *glmer* function from the ‘lme4’ package (Bates et al. 2015). ‘Feeder sites’ was modelled as a binary variable (0 = single feeder site used, 1 = multiple feeder sites used) because the distribution of feeder sites was heavily skewed to single feeder site users (see Figure S4), such that modelling continuous count data led to substantial underdispersion (results not shown).

Both post hoc models had the same fixed effects (age‐ sex, competition treatment, and the interaction of competition treatment and age‐ sex), and random effects (individual ID and trial number). However, we did not include ambient temperature and days since the start of the treatment in these two models, as we were only interested in the interaction between age‐ sex and treatment, and neither temperature nor days since the start of treatment were confounded with age‐ sex. All data and code to reproduce the analyses and figures presented in the main text and ESM are provided here: https://doi.org/10.17605/OSF.IO/2X9YV.

## 3 Results

Over the course of the study, 126 PIT‐tagged chickadees were detected (*N* = 51 adult males, *N* = 49 adult females, *N* = 14 immature males, *N* = 12 immature females) at the experimental feeders on days when both feeders were full (i.e., low competition, the reference category). The daily average temperature ranged from −27.2°C to −2.57°C on days the feeder treatments were in effect, with an average of −11.5°C (±6.7°C s.d.) on treatment days. By chance, there was a significant difference in daily average temperature between the single and two‐feeder treatments (Figure S5): daily average temperature was significantly lower during the two‐feeder (i.e., low competition) treatment (mean daily average temp on two‐feeder days = −14.4°C ± 8.2 s.d.; mean daily average temp on single‐feeder days = −8.6°C ± 3.0 s.d.; *t* = −2.3, *p* = 0.04, df = 13.8). This difference was driven by a rapid increase in temperature from the two‐feeder to single‐feeder per site treatment in the third trial of the study (see Figure S5). On average, chickadees made 124.19 (± 81.71 s.d.) total daily visits to full feeders on feeder treatment days (*N* = 126 birds).

**FIGURE 1:**
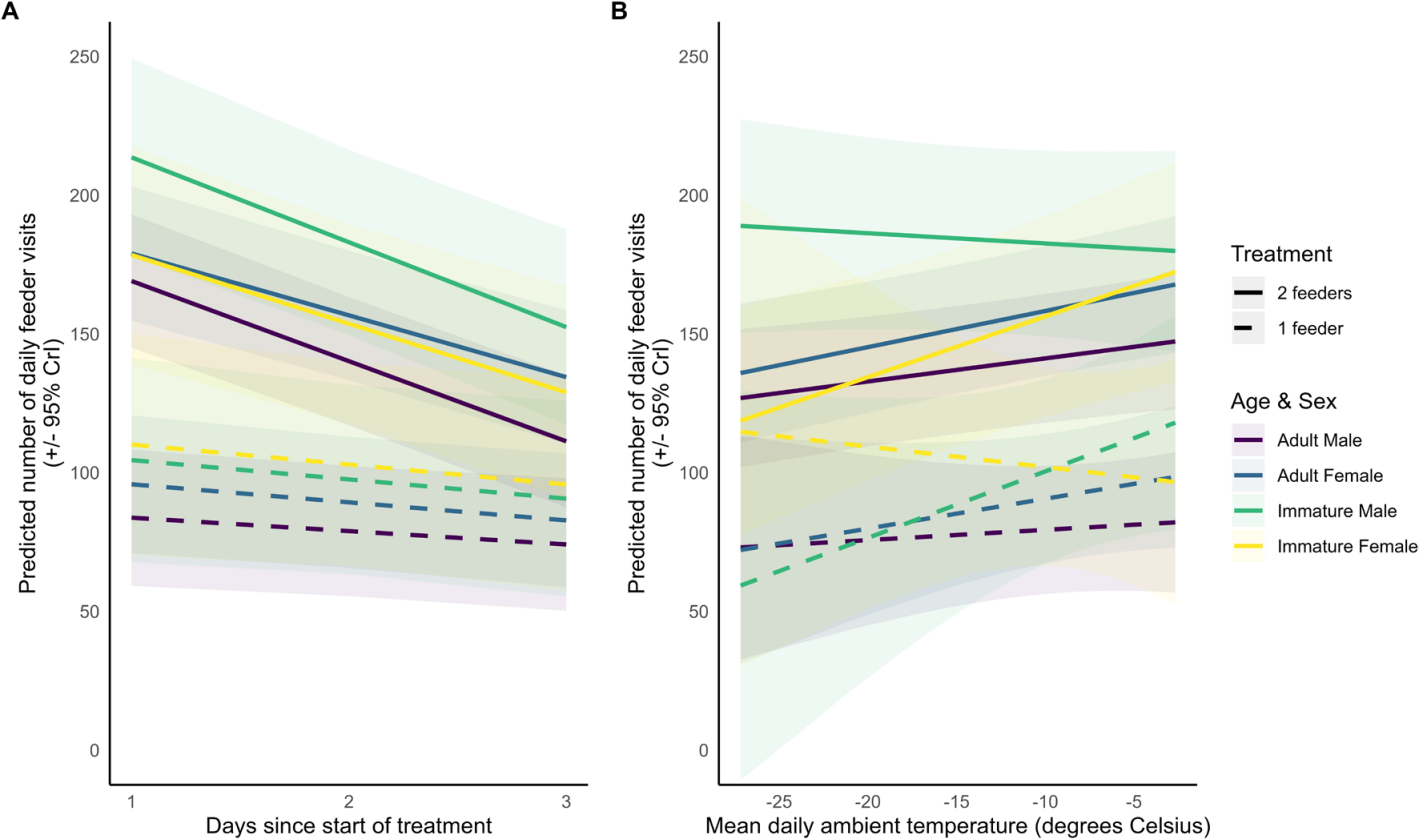
Feeder treatment (two vs. one feeder) effects on per capita daily feeder visits as a function of days since start of treatment (A), and temperature (B). Age‐sex categories are illustrated with different colours, while treatments are illustrated with different line types (see legend). Values plotted are predictions derived from linear models using the ggeffects package (Lüdecke 2018), and shaded areas represent 95% CrIs. Colours were plotted using the viridis package (Garnier et al. 2024).

Overall, daily per capita feeder visits were higher in the two‐feeder treatment than the single‐feeder treatment (Table 1). Daily feeder visits during the two‐feeder treatment were highest in immature males and lowest in adult males, with adult and immature females having intermediate daily feeder visits to the age classes of males (Table 1). Daily number of feeder visits decreased across days during the two‐feeder treatment at similar rates for all age and sex categories. Declines across days during the single‐feeder treatment were significantly shallower compared to the two‐feeder treatment for all age‐sex groups (Table 1, Figure 1A). The total daily feeder visits either increased with increasing mean daily temperature or were not significantly different from 0 (i.e., no slope) for all age‐sex categories and did not differ as a function of feeder treatment (Table 1, Figure 1B).

**TABLE 1:** Simulated posterior means and 95% credible intervals (CrI) for the estimated effect of feeder treatment as a function of temperature, age‐ sex, and days since the start of treatment on total daily feeder visits.

Fixed effects	Adult male	Adult female	Immature male	Immature female
	*β* (95% CrI)	*β* (95% CrI)	*β* (95% CrI)	*β* (95% CrI)
Intercept^a^	163 (145, 192)	180 (153, 200)	218 (180, 251)	185 (143, 218)
Feeders (1)	−86 (−98, −73)	−83 (−96, −70)	−108 (−135, −84)	−62 (−96, −38)
Feeders2:Temp^b^	4 (1–10)	10 (4–13)	−2 (−11, 7)	13 (3, 23)
Feeders1:Temp^b^	4 (−10, 12)	3 (−5, 17)	14 (−5, 36)	−2 (−32, 21)
Feeder2:Days^c^	−29 (−35, −23)	−22 (−29, −16)	−31 (−43, −19)	−24 (−40, −11)
Feeders1:Days^c^	−4 (−11, 1)	−7 (−13, −1)	−6 (−18, 6)	−5 (−21, 5)

Random effects	*σ* (95% CrI)
ID	2514 (2162, 2796)
Trial number	299 (228, 416)
Residual	2979 (2776, 3120)

*Note:* The first treatment was comprised of two feeders filled with sunflower seeds at each of the eight feeder sites, and the second treatment was comprised of one full feeder at each of the eight sites. Daily foraging visits were taken as the sum of visits to all full feeders at the study site, per individual (*N* = 126 individuals, *n* = 2371 observations). Daily average temperature was measured in degrees Celsius. Estimated effects for each age‐sex category are presented in separate columns for ease of comparison, but values presented are derived from a single linear mixed effect model with Gaussian error distribution. Results from the same model fixed effect structure with Poisson error distribution are presented in ESM Table S2.

^a^ Separate intercepts estimated for each age/sex category. Intercept estimates are for the two‐feeder treatment, at the mean daily ambient temperature in the dataset (−11.52°C) and on the first day of the treatment.

^b^ Average daily temperature was measured in degrees Celsius and was centred and standardised prior to analysis. Thus, temperature effects are estimated for a change of 1 SD (6.72°C).

^c^ Days since start of treatment was left zeroed prior to analysis so that the intercept was estimated on Day 1. Estimated effect is change in daily feeder visits per day.

**FIGURE 2:**
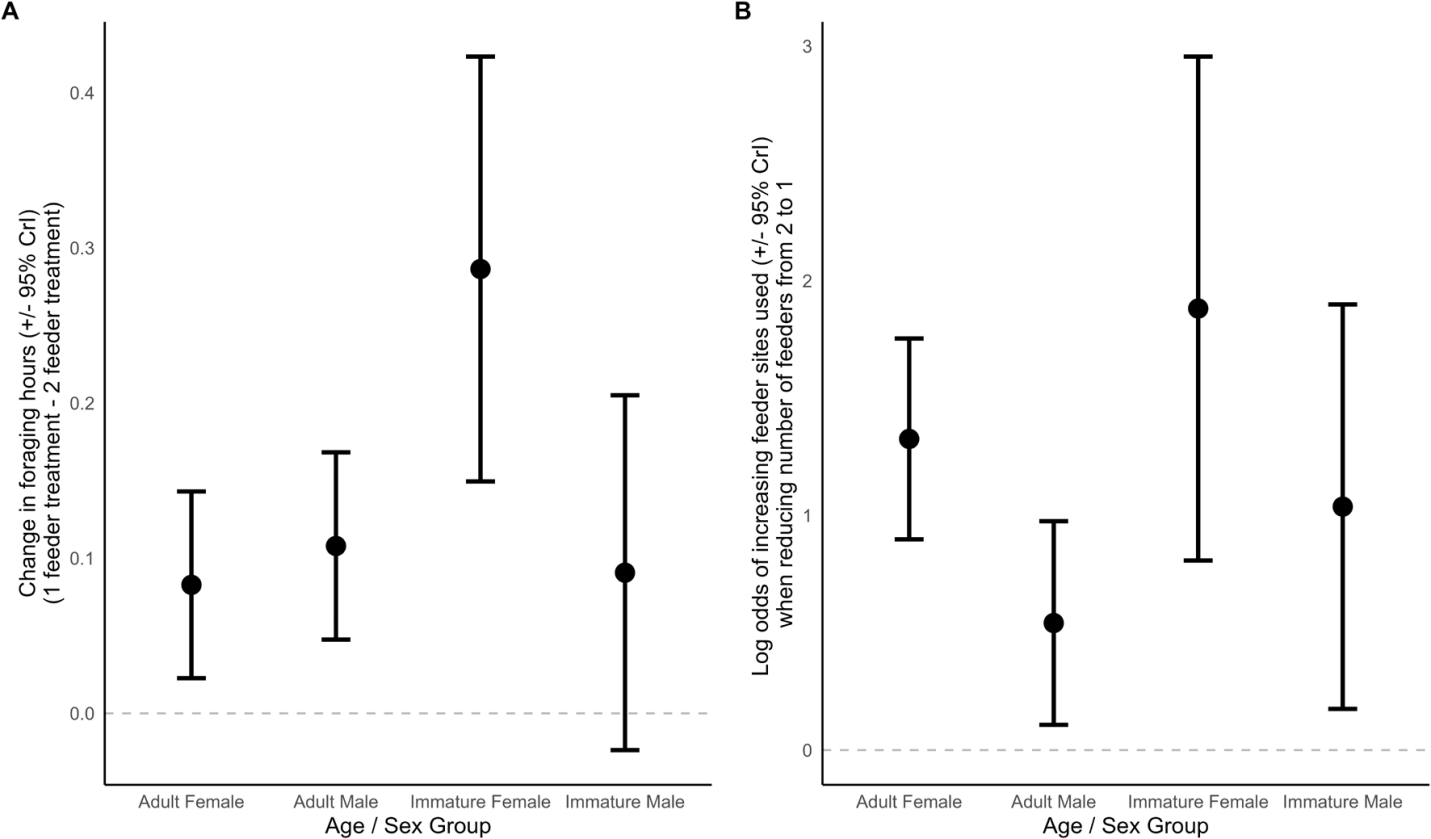
Effect of changing feeder treatment from two to one feeder per feeder site on the total number of foraging hours (of observations where daily foraging hours was within ±2.5 h of daylength) (A) and the log‐odds of increasing the likelihood of using more than one feeder site as a function of age‐sex category (B).

Post hoc analysis on the length of the daily foraging period revealed that the total number of foraging hours increased during the single‐feeder treatment for all age‐sex categories, and total foraging hours were similar for all age‐sex categories during the two‐feeder treatment (i.e., 95% CrIs were broadly overlapping, Table 2). Adult males, adult females, and immature males all responded similarly to decreasing the number of full feeders per site from two to one, however, immature females showed significantly greater increases in foraging hours compared to all other age‐sex categories (Table 3, Figure 2A). Analyses of the probability of using multiple feeder sites again showed broadly similar values for all age‐sex categories during the two‐feeder treatment (Table 2). All age‐sex categories showed significant increases in the probability of using multiple feeder sites when the number of feeders per site was decreased from two to one (Table 2, Figure 2B) and there was strong support for age and sex‐related differences in response to the feeder treatments. Immature females and adult females exhibited the greatest increase, and this increase was significantly greater than that observed in adult males, and moderately greater than that observed in immature males (Table 3, Figure 2B). Adult males had the smallest increase, and there was moderate support that this increase was less than that observed in immature males (Table 3, Figure 2B). Immature males were intermediate to females and adult males.

**TABLE 2:** Simulated posterior means and 95% credible intervals (CrI) for the estimated effect of feeder treatment, age‐ sex, and their interaction on total time spent foraging and the log odds of using more than one feeder site.

Fixed effects	Foraging hours	Feeder sites
	*β* (95% CrI)	*β* (95% CrI)
Adult‐Male	7.67 (7.15, 8.28)	−3.67 (−4.48, −2.69)
Adult‐Female	7.79 (7.32, 8.45)	−3.22 (−4.43, −2.49)
Immature‐Male	8.14 (7.53, 8.72)	−4.01 (−6.23, −2.56)
Immature‐Female	8.08 (7.37, 8.58)	−4.78 (−6.55, −2.53)
Feeders(1)		
Adult‐Male	0.11 (0.04, 0.17)	0.49 (0.11, 0.96)
Adult‐Female	0.10 (0.02, 0.14)	1.38 (0.92, 1.76)
Immature‐Male	0.10 (−0.02, 0.20)	1.19 (0.24, 1.96)
Immature‐Female	0.27 (0.15, 0.43)	1.93 (0.78, 2.94)

Random effects	*σ* (95% CrI)	*σ* (95% CrI)
ID	0.08 (0.07, 0.10)	6.51 (5.24, 7.79)
Trial	0.33 (0.31, 0.36)	0.05 (0.01, 0.12)
Residual	0.20 (0.19, 0.21)	1

*Note:* Foraging hours (continuous) were modelled with Gaussian errors (*N* = 122 individuals, *n* = 2154 observations), while the probability of using more than one feeder site was modelled with binomial errors (binary: 1 site or > 1 site; *N* = 126 individuals, *n* = 2340 observations). Separate intercepts (two‐feeder treatment) and slopes (change from two‐feeder treatment to single‐feeder treatment) were estimated for each age‐sex category during the two‐feeder treatment to facilitate pairwise comparisons between all age‐sex classes.

**TABLE 3:** Pairwise contrasts of treatment effects on total foraging hours (above the diagonal), and probability of using multiple feeder sites (below the diagonal).

	Adult female	Immature female	Adult male	Immature male
Adult female	—	−0.21 (−0.35, −0.05)	−0.04 (−0.12, 0.05) pr = 0.28	−0.03 (−0.14, 0.12)
Immature female	0.45 (−0.59, 1.73) pr = 0.17	—	0.17 (0.03, 0.35)	0.16 (0.01, 0.38)
Adult male	−0.70 (−1.49, −0.27)	−0.97 (−2.37, −0.13)	—	0.01 (−0.10, 0.16)
Immature male	−0.29 (−1.25, 0.71) pr = 0.26	−0.81 (−2.31, 0.42) pr = 0.11	0.21 (−0.51, 1.43) pr = 0.11	—

*Note:* Values are the posterior mode of the differences between age‐sex (category in left column minus category in top row) and 95% CrI, calculated from the models presented in Table 2.

## 4 Discussion

We experimentally manipulated food availability in a free‐living population of chickadees to evaluate support for interference competition in this system. We found strong support that reducing food availability, from two ad libitum feeders to one ad libitum feeder per site, resulted in an overall reduction in foraging rates at the population level, consistent with interference competition limiting access to feeders. Although males are dominant over females in chickadees (Desrochers 1989; Desrochers et al. 1988; Smith 1976), and at least in some populations of chickadees, adults are dominant over immatures (Desrochers 1989; Desrochers et al. 1988), we found no evidence that females and/or immatures experienced greater interference competition. Post hoc analyses suggest that females (both adult and immature) may have mitigated interference competition by expanding their foraging area, while immature females additionally increased foraging hours under increased competition. We discuss these results in light of sex‐related differences in the importance of uncertainty mitigation strategies.

Consistent with earlier empirical (Cresswell 1998; Goss‐Custard 1980) and theoretical work (Ydenberg et al. 1986), we found that manipulating food availability resulted in changes in total daily feeder visits, a proxy for food acquisition. Specifically, reducing the number of available feeders led to reductions in daily feeder visits, even though food was provided ad libitum and therefore not limited in both treatments. We found that the increase in competition brought about by halving the number of feeders available per site resulted in similar reductions in per capita feeder visits for all age and sex classes: circa 50%. Previous work in chickadees has shown that the average visit duration to feeders baited with sunflower seeds is 2 s (Hughes et al. 2021). Thus, given the number of birds using the feeders combined with total daily feeder visits, reducing the number of feeders per site from two to one cannot explain the reduction in feeding rates in the absence of interference competition, as there was clearly sufficient time to allow chickadees to maintain the same total daily feeder visits in the single‐feeder as they had during the two‐ feeder treatment. Such reductions in daily feeder visits with decreasing feeder density are consistent with an increase in interference competition limiting access to feeders in chickadees, such as via increased time spent interacting with or observing conspecifics.

An alternative interpretation of our finding that daily feeder visits decreased under increased competition (i.e., when a single feeder was available) is that it is an artefact of the fixed treatment order, rather than treatment per se. Specifically, as the two‐feeder (low competition) treatments were always preceded by 12 days without food, chickadees may have been expected to deplete their caches during the period without supplemental food. As such, upon rediscovery of feeders that were full, chickadees might initially have high feeder visit rates as they work to rebuild their caches, with daily feeder visits decreasing as caches are replenished. Higher levels of caching initially upon feeder rediscovery could also be expected based on theoretical models which predict that caching rates should vary inversely with food predictability (Brodin and Clark 1997; Hitchcock and Houston 1994; Lucas and Walter 1991; McNamara et al. 1990), such that pulsed food availability promotes initially high levels of caching which decline with increasing number of days of predictable access to feeders. Indeed, we observed that total daily feeder visits declined during the two‐feeder treatment as a function of days since the treatment start. As initial feeder visits (range 161–209 between age/sex classes) far exceeded the expected daily energy requirements (estimated to be 70 seeds per day when ambient temperature is ~−10°C) (Karasov et al. 1992; Lajoie et al. 2019), this is consistent with high levels of caching, which declined across days. However, importantly, the effect of days since start of treatment during the single‐feeder (high competition) treatment was either not significantly different from zero (immature females, adult males, immature males), or was small and significantly lower compared to the effect during the two‐feeder treatment (adult females), suggesting a lower, but relatively stable, level of feeder use under single‐feeder conditions (Figure 1A). Thus, we conclude that although the pulsed resources may have favoured high levels of caching in our study, temporal trends in caching alone cannot explain the decrease in total daily feeder visits under experimentally increased competition.

We also observed an overall tendency for a positive relationship between total daily feeder visits and daily average ambient temperature during the two‐feeder treatment, and no significant relationship between total daily feeder visits and daily average ambient temperature during the single‐feeder treatment (Figure 1B). Positive relationships are *contra* to predictions based on the fact that energetic demands increase with decreasing temperatures due to the increased costs of thermoregulation, which should lead to higher foraging rates at lower ambient temperatures (Broggi et al. 2004). Indeed, numerous earlier studies have found that black‐capped chickadees increase daily foraging rates with decreasing temperatures (Arteaga‐Torres et al. 2020; Bonter et al. 2013; Hawkshaw et al. 2025; Hobbs et al. 2024; Kessel 1976; LaRocque et al. 2024; Latimer et al. 2018). We are aware of only two studies in black‐capped chickadees that found no support that feeder use increased with decreasing ambient temperature (Lajoie et al. 2019; Wilson 2001). The study by Lajoie et al. (2019) occurred in a Mediterranean climate (Corvallis, OR, 44°33′ N, 123°15′ W), and although temperature data were not provided, the environmental conditions were described as mild, with ‘no regular snow cover and temperatures above freezing’. Thus, the range of temperatures in that study was almost completely non‐overlapping with the temperatures in other studies which did report a negative relationship between feeder visits and ambient temperature while providing continuous access to food. This may indicate that relationships between feeder use and ambient temperature are non‐linear. In the study by Wilson (2001), no significant negative relationship between ambient temperature and feeder use was observed. However, this study may have lacked statistical power to detect temperature effects as the total sample sizes used to evaluate relationships between feeder use and ambient temperature at each of two feeder sites tested were *n* = 51 and *n* = 47. Given that there were 13 sampling days, this implies an average of 3.9 and 3.6 individuals respectively used to estimate feeder visits for each day in the correlation tests with ambient temperature, which is unlikely to be sufficient power given high individual variation in feeder use reported in that study (total seeds removed during observations per bird ranged from 2 to 175; Wilson 2001). Thus, the general pattern for studies conducted at ambient temperatures below freezing is that chickadees increase total daily feeder visits with decreasing ambient temperatures. However, the total foraging rates observed in the above studies with continuously available food were generally close to the expected daily energy requirements of birds, suggesting a limited role of caching. We suggest that this discrepancy between earlier studies and the present study in terms of the relationship between daily feeder visits and ambient temperature may be explained by different responses by chickadees to continuous versus pulsed supplemental resources. When resources are provided continuously, investment in caching is expected to be reduced (Brodin and Clark 1997; Hitchcock and Houston 1994; Lucas and Walter 1991; McNamara et al. 1990). This may result in daily feeder visits more closely matching daily intake, such that higher total daily feeder visits occur under colder, more energetically challenging conditions. In contrast, when resources are pulsed, and investment in caching increases, daily feeder visits reflect investment in both feeding and caching, with greater investment in caching occurring under less energetically challenging conditions (e.g., Brodin et al. 2001; i.e., milder ambient temperatures).

Given that reducing the number of feeders available per site would increase interference competition, we were interested in evaluating support for the prediction derived from theoretical models that dominant individuals should be less strongly affected by interference competition compared with subordinates (Stillman, Goss‐Custard, and Caldow 1997; Ydenberg et al. 1986). Although males are consistently dominant over females in chickadees (Desrochers 1989; Desrochers et al. 1988; Smith 1976), we found no support for sex‐related differences in total daily feeder visits, nor sex‐related differences in the response to feeder treatments (Figure 1). This result is in contrast to numerous other empirical studies in birds that have found subordinates suffer more from interference competition compared to dominants (Alatalo 1981; Bijleveld et al. 2012; Langen and Rabenold 1994; Radford and Du Plessis 2003; Rutten, Oosterbeek, van der Meer, et al. 2010; Rutten, Oosterbeek, Verhulst, et al. 2010; Vahl and Kingma 2007). Similarly, there was no evidence that immature birds experienced stronger interference competition. This could indicate that immature birds are not subordinate to adult birds in our population, as evidence for age‐related differences in dominance in black‐capped chickadees is variable, with some studies reporting adults are dominant to yearlings (Desrochers 1989; Desrochers et al. 1988), some studies reporting age‐related differences in dominance in males only (Ramsay and Ratcliffe 2003; Schubert et al. 2007; Smith 1976), and other studies reporting no age effects on dominance (Brittingham and Temple 1992; Wilson 2001). However, changes in spatial and temporal patterns of foraging in immature birds in response to the decrease in number of ad libitum feeders available per site suggest that immature birds are likely subordinate to adults in our population (see below).

If females and immatures are subordinate to males and adults in black‐capped chickadees, this begs the question—why did we not observe age and/or sex‐related differences in daily feeder visits in response to reducing the number of feeders per site? One possibility is that females and/or immatures adjusted other aspects of their behaviour in ways that allowed them to achieve similar feeder visit rates to males and/or adults despite their subordinate status. For example, subordinates may increase their access to food by extending their foraging window (Krams 2000), foraging at riskier times (Alanärä et al. 2002; Lahti et al. 1997) and/or in riskier places (Barluenga et al. 2008; Ekman 1987; Koivula et al. 1994; Schneider 1984; Slotow and Paxinos 1997). Therefore, we conducted post hoc tests to evaluate whether females and/or immatures extended the length of their foraging windows compared to males and/or adults, or whether they foraged across a larger spatial scale. We found that the single‐feeder treatment did increase the length of the foraging window in chickadees. However, this effect was similar for males (both adult and immature) and adult females (~7 min, Tables 2 and 3). Immature females, however, increased the duration of foraging significantly more (~30 min) compared to all other age‐sex categories during the single‐feeder treatment (Tables 2 and 3, Figure 2A). Similar results have been observed in response to interspecific competition; as the number of great tits ( *Parus major* ) and blue tits ( *Cyanistes caeruleus* ) using a feeder site increased, marsh tits ( *Poecile palustris* ), which are subordinate to both great tits and blue tits, exhibited temporal avoidance by concentrating their use of feeders early in the day before peaks in activity by great tits and blue tits (Maziarz et al. 2023). By extending the foraging window, immature female chickadees may similarly gain some temporal refuge from interference competition, though it would also be interesting to investigate whether they exhibit temporal avoidance on finer scales.

We also found that all age‐sex classes had a significant increase in the likelihood of using multiple feeder sites during the single‐feeder treatment (Table 2). However, females (both adults and immatures) increased space use significantly more than adult males, while immature males exhibited increased space use that was intermediate to the other age/sex classes (Table 3 and Figure 2B). This is similar to results observed in mallards (*Anas platyrhynochos*) where subordinates were able to achieve similar intake rates as dominants, and did so by using a larger number of food patches (Gyimesi et al. 2010). Thus, taken together, these results suggest that immature females may have mitigated competition by expanding their foraging in both time and space, while adult females appeared to only expand foraging in space, relative to adult males, the dominant age‐sex class. There was also moderate support that immature males increased space use more than adult males. Overall, these patterns are consistent with males being dominant over females, and adults being dominant over subordinates.

The observation that females and immatures did not experience larger reductions in feeding rate compared to males and adults in response to increased competition suggests that the costs of increased time spent foraging and/or travel costs associated with foraging across more feeder sites may have been offset by the benefits of maintaining feeder visit rates. Again, we suggest that the pulsed nature of resource supplementation in our study may have favoured strategies to mitigate uncertainty in food availability, particularly in females and immatures, for whom uncertainty should be more consequential due to their subordinate status (Dall 2010). As previously noted, the levels of total daily feeder use reported in the present study substantially exceeded the daily intake requirements of chickadees, suggesting significant levels of caching were occurring throughout the study. Previous work in chickadees has shown that females invested more in updating their information about feeders by regularly sampling empty feeders (Haave‐Audet et al. 2024). It would be informative to conduct a study that similarly alternated between low (two feeders per site) and high (one feeder per site) competition treatments, but additionally manipulated whether there were periods without food between treatment replicates in order to directly evaluate the importance of pulsed versus constant food in shaping sex‐specific patterns in response to increased competition.

In conclusion, our study provides strong evidence that interference competition limits access to feeders in free‐living chickadees. Reducing the number of ad libitum feeders available by half led to a *circa* 50% reduction in per capita daily feeder visit rates. While there was no evidence for sex‐ or age‐related differences in competition induced reductions in feeding rates, subordinate classes appeared to achieve this by expanding the time spent foraging and/or their foraging area more than the dominant age‐sex class (adult males) under the increased competition treatment. Several of our results suggest that the pulsed nature of food supplementation may have had important consequences for chickadees, particularly in terms of investment in caching behaviour. Future studies that simultaneously manipulate competition level (e.g., one versus two feeders per site) and predictability (e.g., pulsed versus continuous food supplementation) are needed to tease apart the relative importance of interference competition from uncertainty management in shaping foraging decisions in chickadees.

## Author Contributions

**Elène Haave‐Audet:** conceptualization (equal), data curation (lead), formal analysis (equal), funding acquisition (supporting), investigation (lead), visualization (equal), writing – original draft (equal), writing – review and editing (equal). **Kimberley J. Mathot:** conceptualization (equal), formal analysis (equal), funding acquisition (lead), investigation (supporting), project administration (lead), supervision (lead), visualization (equal), writing – original draft (equal), writing – review and editing (equal).

## Funding

E.H.‐A. was supported by the Natural Sciences and Engineering Research Council (NSERC) Canada Graduate Scholarships Master's program and the Alberta Graduate Excellence Scholarship. Fieldwork for this study was supported by a grant from the Alberta Conservation Association Grants in Biodiversity program to E.H.‐A. and by University of Alberta start‐up funds, Canda Research Chair research funds, and an NSERC Discovery Grant to (RGPIN‐2018‐04358) to K.J.M.

## Conflicts of Interest

The authors declare no conflicts of interest.

## Supporting information


**Table S1:** Detailed timeline of the entire experiment. Feeder status indicates whether one, both, or neither feeder at each feeder site was full. Competition treatment indicates the conditions we expected based on food availability (Low competition = two ad libitum feeders per site; High competition = one ad libitum feeder per site). ‘Total daily feeder visits counted’ indicates whether daily foraging rates were included in the analyses on a given day. We excluded days on which treatments were changed (days since start of treatment = 0), because filling/emptying feeders occurred partway through the day, meaning that the treatment did not apply to the full foraging day, precluding us from calculating total daily feeder visits.
**Table S2:** Simulated posterior means and 95% credible intervals for the estimated effect of feeder treatment as a function of temperature, age‐ sex, days since the start of treatment on total daily feeder visits. The first treatment was comprised of two feeders filled with sunflower seeds at each of the eight feeder sites, and the second treatment was comprised of one full feeder at each of the eight sites. Daily foraging visits were taken as the sum of visits to all full feeders at the study site, per individual. Daily average temperature was measured in degrees Celsius. Estimated effects for each age‐ sex category are presented in separate columns for ease of comparison, but values presented are derived from a single generalised linear mixed effect model with Poisson error distribution. Results from the same model fixed effect structure with Gaussian error distribution is presented in the main text (Table 1).
**Table S3:** Simulated posterior means and 95% credible intervals for the estimated effect of feeder treatment, age‐ sex, and their interaction on total time spent foraging modelled with Gaussian errors. Separate intercepts (two‐feeder treatment) and slopes (change from two‐feeder treatment to single‐feeder treatment) were estimated for each age‐sex category during the two‐feeder treatment to facilitate pairwise comparisons between all age‐sex classes. Analyses in main text removed birds with low overall feeder visits to improve model fit. Analysis presented here includes all birds and is qualitatively similar to results presented in main text Table 2.
**Table S4:** Simulated posterior means and 95% credible intervals for the estimated effect of feeder treatment, age‐ sex, and their interaction on total daily feeder visits modelled with Gaussian errors, for daily bird observations with a foraging window within ±2.5 h of daylength. Separate intercepts (two‐feeder treatment) and slopes (change from two‐feeder treatment to single‐feeder treatment) were estimated for each age‐sex category during the two‐feeder treatment to facilitate pairwise comparisons between all age‐sex classes. Analyses in main text included all observations. Analysis presented here is qualitatively similar to results presented in main text Table 1.
**Figure S1:** Photo showing feeders used in the study. The copper circle is the RFID antenna, which provides a perch for birds to sit on while accessing sunflower seeds from a small opening behind it (3 cm diameter). The hinges visible on the right lower side of the feeder open a compartment where the RFID board with SD card and battery are located. The back and top of the feeder provide a large reservoir of sunflower seeds such that the feeders do not become depleted between successive observer visits (every four days). Feeders are protected in wire mesh to prevent destruction by squirrels. The chicken wire is used to restrict access to the area where sunflower seeds are available by non‐focal species, but allows chickadees to pass through freely.
**Figure S2:** Histogram of the count of daily feeder visits per bird.
**Figure S3:** Histogram of difference between total daylength (hours from sunrise to sunset) and foraging hours (hours between first and last feeder detection per bird per day). Most birds have foraging lengths within ±2.5 h of daylength, with a long tail of longer differences. These birds had very short foraging windows at feeders, suggesting they were not heavily reliant on feeders.
**Figure S4:** Histogram of the count of feeder sites used per bird per day. Values were heavily skewed towards 1, therefore, data were modelled as binary (1 site used or > 1 site used).
**Figure S5:** Daily average temperature over the course of the competition experiment. Low (two feeders per site) and high (one feeder per site) competition treatment days are labelled, and grey shading indicates periods at which time data were not collected for this study. By chance, there was a significant temperature drop during the third trial of the low competition treatment, driving a significant difference in average temperatures between low and high competition conditions.
**Text S1:** Investigating model fit.

## Data Availability

Data and code required to reproduce all analyses and figures presented in the main text is publicly available from the Open Science Framework (OSF) repository (https://doi.org/10.17605/OSF.IO/2X9YV).
