# Supplementary material for: Female Black‐Capped Chickadees (Poecile atricapillus) Expand Foraging Both Temporally and Spatially to Mitigate Interference Competition
Source: Ecol Evol. 2026 Jul 10;16(7):e73972. doi: 10.1002/ece3.73972 (PMC13354752; doi:10.1002/ece3.73972)
Supplement: Supplementary file 1 — Table S1: Detailed timeline of the entire experiment. Feeder status indicates whether one, both, or neither feeder at each feeder site was full. Competition treatment indicates the conditions we expected based on food availability (Low competition = two ad libitum feeders per site; High competition = one ad libitum feeder per site). ‘Total daily feeder visits counted’ indicates whether daily foraging rates were included in the analyses on a given day. We excluded days on which treatments were changed (days since start of treatment = 0), because filling/emptying feeders occurred partway through the day, meaning that the treatment did not apply to the full foraging day, precluding us from calculating total daily feeder visits. Table S2: Simulated posterior means and 95% credible intervals for the estimated effect of feeder treatment as a function of temperature, age‐ sex, days since the start of treatment on total daily feeder visits. The first treatment was comprised of two feeders filled with sunflower seeds at each of the eight feeder sites, and the second treatment was comprised of one full feeder at each of the eight sites. Daily foraging visits were taken as the sum of visits to all full feeders at the study site, per individual. Daily average temperature was measured in degrees Celsius. Estimated effects for each age‐ sex category are presented in separate columns for ease of comparison, but values presented are derived from a single generalised linear mixed effect model with Poisson error distribution. Results from the same model fixed effect structure with Gaussian error distribution is presented in the main text (Table 1). Table S3: Simulated posterior means and 95% credible intervals for the estimated effect of feeder treatment, age‐ sex, and their interaction on total time spent foraging modelled with Gaussian errors. Separate intercepts (two‐feeder treatment) and slopes (change from two‐feeder treatment to single‐feeder treatment) were estimated for each [file ECE3-16-e73972-s001.docx]

**Supplementary material for**: Female black-capped chickadees (*Poecile atricapillus*) expand foraging both temporally and spatially to mitigate interference competition

Authors: Elène Haave-Audet and Kimberley J. Mathot

Contents

[Text S1: Investigating model fit 3](#_Toc233967605)

[Table S1. Detailed timeline of the entire experiment. Feeder status indicates whether one, both, or neither feeder at each feeder site was full. Competition treatment indicates the conditions we expected based on food availability (Low competition = 2 ad libitum feeders per site; High competition = 1 ad libitum feeder per site). ‘Total daily feeder visits counted’ indicates whether daily foraging rates were included in the analyses on a given day. We excluded days on which treatments were changed (days since start of treatment = 0), because filling/emptying feeders occurred partway through the day, meaning that the treatment did not apply to the full foraging day, precluding us from calculating total daily feeder visits. 4](#_Toc233967606)

[Table S2. Simulated posterior means and 95% credible intervals for the estimated effect of feeder treatment as a function of temperature, AgeSex, days since the start of treatment on total daily feeder visits. The first treatment was comprised of two feeders filled with sunflower seeds at each of the eight feeder sites, and the second treatment was comprised of one full feeder at each of the eight sites. Daily foraging visits were taken as the sum of visits to all full feeders at the study site, per individual. Daily average temperature was measured in degrees Celsius. Estimated effects for each AgeSex category are presented in separate columns for ease of comparison, but values presented are derived from a single generalized linear mixed effect model with Poisson error distribution. Results from the same model fixed effect structure with Gaussian error distribution is presented in the main text (Table 1). 7](#_Toc233967607)

[Table S3. Simulated posterior means and 95% credible intervals for the estimated effect of feeder treatment, AgeSex, and their interaction on total time spent foraging modelled with Gaussian errors. Separate intercepts (two-feeder treatment) and slopes (change from two-feeder treatment to single-feeder treatment) were estimated for each Age-Sex category during the two-feeder treatment to facilitate pairwise comparisons between all age-sex classes. Analyses in main text removed birds with low overall feeder visits to improve model fit. Analysis presented here includes all birds and is qualitatively similar to results presented in main text Table 2. 8](#_Toc233967608)

[Table S4. Simulated posterior means and 95% credible intervals for the estimated effect of feeder treatment, AgeSex, and their interaction on total daily feeder visits modelled with Gaussian errors, for daily bird observations with a foraging window within +/- 2.5 hrs of daylength. Separate intercepts (two-feeder treatment) and slopes (change from two-feeder treatment to single-feeder treatment) were estimated for each AgeSex category during the two-feeder treatment to facilitate pairwise comparisons between all age-sex classes. Analyses in main text included all observations. Analysis presented here is qualitatively similar to results presented in main text Table 1. 9](#_Toc233967609)

[Figure S1: Photo showing feeders used in the study. The copper circle is the RFID antenna, which provides a perch for birds to sit on while accessing sunflower seeds from a small opening behind it (3 cm diameter). The hinges visible on the right lower side of the feeder open a compartment where the RFID board with SD card and battery are located. The back and top of the feeder provide a large reservoir of sunflower seeds such that the feeders do not become depleted between successive observer visits (every 4 days). Feeders are protected in wire mesh to prevent destruction by squirrels. The chicken wire is used to restrict access to the area where sunflower seeds are available by non-focal species, but allows chickadees to pass through freely. 10](#_Toc233967610)

[Figure S2: Histogram of the count of daily feeder visits per bird. 11](#_Toc233967611)

[Figure S3: Histogram of difference between total daylength (hours from sunrise to sunset) and foraging hours (hours between first and last feeder detection per bird per day). Most birds have foraging lengths within +/- 2.5 hours of daylength, with a long tail of longer differences. These birds had very short foraging windows at feeders, suggesting they were not heavily reliant on feeders. 12](#_Toc233967612)

[Figure S4: Histogram of the count of feeder sites used per bird per day. Values were heavily skewed towards 1, therefore, data were modelled as binary (1 site used or >1 site used). 13](#_Toc233967613)

[Figure S5. Daily average temperature over the course of the competition experiment. Low (two feeders per site) and high (one feeder per site) competition treatment days are labelled, and grey shading indicates periods at which time data were not collected for this study. By chance, there was a significant temperature drop during the third round of the low competition treatment, driving a significant difference in average temperatures between low and high competition conditions. 14](#_Toc233967614)

# ****Text S1: Investigating model fit****

For models presented in the main text, we first verified that models achieved good fit. When they did not, we evaluated alternative model structures to ensure that the inferences drawn from models presented in the main text were robust across alternative model specifications. All alternative models were constructed with the same fixed and random effects as presented in the main text but were modelled with different error families (e.g., Poisson).

The model investigating the daily count of visits was modelled with an untransformed response variable and Gaussian error distributions in the main text (main text Table 1). Model residuals were normally distributed, but plots of fitted values against residuals indicated some heteroscedasticity. As the data for total daily feeder visits were Poisson distributed (see ESM Figure S1), we verified that model inferences were the same when data were modelled with Poisson errors, which they were (ESM Table S2). However, the Poisson model was overdispersed, so we modelled residual variance by including an observation-level random effect. We presented the untransformed Gaussian model in the main text for ease of interpretation.

The models investigating total foraging hours satisfied model assumptions, thus we only present results of the untransformed linear models. However, analyses in main text filtered out birds with low total foraging hours suggestive that they relied on other food sources. We present analyses in the ESM on unfiltered data to show that patterns were qualitatively similar (Table S3) to those presented in the main text (Table 2).

The data for total daily feeder sites were heavily skewed; given that birds were present at feeders, on most occasions, birds used a single feeder site, with rare occurrences of using two or more feeder sites (see EMS Figure S2). We therefore treated this as a binary variable where observations corresponding to a single feeder site were coded as 0, and observations corresponding to two or more feeder sites were coded as 1. This binary response variable was modelled with binomial errors, and thus, model output is the log odds ratio of increasing the likelihood of using > 1 feeder site. The model was slightly underdispersed, which increases the likelihood of making a Type II error (i.e., fail to detect an effect when it exists), meaning that the interpretations derived from underdispersed models are more conservative.

# **Table S1.** **Detailed timeline of the entire experiment**. Feeder status indicates whether one, both, or neither feeder at each feeder site was full. Competition treatment indicates the conditions we expected based on food availability (Low competition = 2 ad libitum feeders per site; High competition = 1 ad libitum feeder per site). ‘Total daily feeder visits counted’ indicates whether daily foraging rates were included in the analyses on a given day. We excluded days on which treatments were changed (days since start of treatment = 0), because filling/emptying feeders occurred partway through the day, meaning that the treatment did not apply to the full foraging day, precluding us from calculating total daily feeder visits.

| Day | Feeder Status | Competition Treatment | Trial # | Total daily feeder visits counted | Days since start of treatment | Battery Change |
| --- | --- | --- | --- | --- | --- | --- |
| Nov 29, 2019 | Full-Full | Low competition | 1 | No | 0 | X |
| Nov 30, 2019 | Full-Full | Low competition | 1 | Yes | 1 |  |
| Dec 1, 2019 | Full-Full | Low competition | 1 | Yes | 2 |  |
| Dec 2, 2019 | Full-Full | Low competition | 1 | Yes | 3 |  |
| Dec 3, 2019 | Full-Empty | High competition | 1 | No | 0 | X |
| Dec 4, 2019 | Full-Empty | High competition | 1 | Yes | 1 |  |
| Dec 5, 2019 | Full-Empty | High competition | 1 | Yes | 2 |  |
| Dec 6, 2019 | Full-Empty | High competition | 1 | Yes | 3 |  |
| Dec 7, 2019 | Empty-Full | N/A | 1 | No | N/A | X |
| Dec 8, 2019 | Empty-Full | N/A | 1 | No | N/A |  |
| Dec 9, 2019 | Empty-Full | N/A | 1 | No | N/A |  |
| Dec 10, 2019 | Empty-Full | N/A | 1 | No | N/A |  |
| Dec 11, 2019 | Empty-Empty | N/A | 1 | No | N/A | X |
| Dec 12, 2019 | Empty-Empty | N/A | 1 | No | N/A |  |
| Dec 13, 2019 | Empty-Empty | N/A | 1 | No | N/A |  |
| Dec 14, 2019 | Empty-Empty | N/A | 1 | No | N/A |  |
| Dec 15, 2019 | Empty-Empty | N/A | 1 | No | N/A | X |
| Dec 16, 2019 | Empty-Empty | N/A | 1 | No | N/A |  |
| Dec 17, 2019 | Empty-Empty | N/A | 1 | No | N/A |  |
| Dec 18, 2019 | Empty-Empty | N/A | 1 | No | N/A |  |
| Dec 19, 2019 | Empty-Empty | N/A | 1 | No | N/A | X |
| Dec 20, 2019 | Empty-Empty | N/A | 1 | No | N/A |  |
| Dec 21, 2019 | Empty-Empty | N/A | 1 | No | N/A |  |
| Dec 22, 2019 | Empty-Empty | N/A | 1 | No | N/A |  |
| Dec 23, 2019 | Full-Full | Low competition | 2 | No | 0 | X |
| Dec 24, 2019 | Full-Full | Low competition | 2 | Yes | 1 |  |
| Dec 25, 2019 | Full-Full | Low competition | 2 | Yes | 2 |  |
| Dec 26, 2019 | Full-Full | Low competition | 2 | Yes | 3 |  |
| Dec 27, 2019 | Full-Empty | High competition | 2 | No | 0 | X |
| Dec 28, 2019 | Full-Empty | High competition | 2 | Yes | 1 |  |
| Dec 29, 2019 | Full-Empty | High competition | 2 | Yes | 2 |  |
| Dec 30, 2019 | Full-Empty | High competition | 2 | Yes | 3 |  |
| Dec 31, 2019 | Empty-Full | N/A | 2 | No |  | X |
| Jan 1, 2020 | Empty-Full | N/A | 2 | No | N/A |  |
| Jan 2, 2020 | Empty-Full | N/A | 2 | No | N/A |  |
| Jan 3, 2020 | Empty-Full | N/A | 2 | No | N/A |  |
| Jan 4, 2020 | Empty-Empty | N/A | 2 | No | N/A | X |
| Jan 5, 2020 | Empty-Empty | N/A | 2 | No | N/A |  |
| Jan 6, 2020 | Empty-Empty | N/A | 2 | No | N/A |  |
| Jan 7, 2020 | Empty-Empty | N/A | 2 | No | N/A |  |
| Jan 8, 2020 | Empty-Empty | N/A | 2 | No | N/A | X |
| Jan 9, 2020 | Empty-Empty | N/A | 2 | No | N/A |  |
| Jan 10, 2020 | Empty-Empty | N/A | 2 | No | N/A |  |
| Jan 11, 2020 | Empty-Empty | N/A | 2 | No | N/A |  |
| Jan 12, 2020 | Empty-Empty | N/A | 2 | No | N/A | X |
| Jan 13, 2020 | Empty-Empty | N/A | 2 | No | N/A |  |
| Jan 14, 2020 | Empty-Empty | N/A | 2 | No | N/A |  |
| Jan 15, 2020 | Empty-Empty | N/A | 2 | No | N/A |  |
| Jan 16, 2020 | Full-Full | Low competition | 3 | No | 0 | X |
| Jan 17, 2020 | Full-Full | Low competition | 3 | Yes | 1 |  |
| Jan 18 2020 | Full-Full | Low competition | 3 | Yes | 2 |  |
| Jan 19, 2020 | Full-Full | Low competition | 3 | Yes | 3 |  |
| Jan 20, 2020 | Full-Empty | High competition | 3 | No | 0 | X |
| Jan 21, 2020 | Full-Empty | High competition | 3 | Yes | 1 |  |
| Jan 22, 2020 | Full-Empty | High competition | 3 | Yes | 2 |  |
| Jan 23, 2020 | Full-Empty | High competition | 3 | Yes | 3 |  |
| Jan 24, 2020 | Empty-Full | N/A | 3 | No | N/A | X |
| Jan 25, 2020 | Empty-Full | N/A | 3 | No | N/A |  |
| Jan 26, 2020 | Empty-Full | N/A | 3 | No | N/A |  |
| Jan 27, 2020 | Empty-Full | N/A | 3 | No | N/A |  |
| Jan 28, 2020 | Empty-Empty | N/A | 3 | No | N/A | X |
| Jan 29, 2020 | Empty-Empty | N/A | 3 | No | N/A |  |
| Jan 30, 2020 | Empty-Empty | N/A | 3 | No | N/A |  |
| Jan 31, 2020 | Empty-Empty | N/A | 3 | No | N/A |  |
| Feb 1, 2020 | Empty-Empty | N/A | 3 | No | N/A | X |
| Feb 2, 2020 | Empty-Empty | N/A | 3 | No | N/A |  |
| Feb 3, 2020 | Empty-Empty | N/A | 3 | No | N/A |  |
| Feb 4, 2020 | Empty-Empty | N/A | 3 | No | N/A |  |
| Feb 5, 2020 | Empty-Empty | N/A | 3 | No | N/A | X |
| Feb 6, 2020 | Empty-Empty | N/A | 3 | No | N/A |  |
| Feb 7, 2020 | Empty-Empty | N/A | 3 | No | N/A |  |
| Feb 8 2020 | Empty-Empty | N/A | 3 | No | N/A |  |
| Feb 9, 2020 | Full-Full | Low competition | 4 | No | 0 | X |
| Feb 10, 2020 | Full-Full | Low competition | 4 | Yes | 1 |  |
| Feb 11, 2020 | Full-Full | Low competition | 4 | Yes | 2 |  |
| Feb 12, 2020 | Full-Full | Low competition | 4 | Yes | 3 |  |
| Feb 13, 2020 | Full-Empty | High competition | 4 | No | 0 | X |
| Feb 14, 2020 | Full-Empty | High competition | 4 | Yes | 1 |  |
| Feb 15, 2020 | Full-Empty | High competition | 4 | Yes | 2 |  |
| Feb 16, 2020 | Full-Empty | High competition | 4 | Yes | 3 |  |
| Feb 17, 2020 | Empty-Full | N/A | 4 | No | N/A | X |
| Feb 18, 2020 | Empty-Full | N/A | 4 | No | N/A |  |
| Feb 19, 2020 | Empty-Full | N/A | 4 | No | N/A |  |
| Feb 20, 2020 | Empty-Full | N/A | 4 | No | N/A |  |
| Feb 21, 2020 | Empty-Empty | N/A | 4 | No | N/A | X |
| Feb 22, 2020 | Empty-Empty | N/A | 4 | No | N/A |  |
| Feb 23, 2020 | Empty-Empty | N/A | 4 | No | N/A |  |
| Feb 24, 2020 | Empty-Empty | N/A | 4 | No | N/A |  |
| Feb 25, 2020 | Empty-Empty | N/A | 4 | No | N/A | X |
| Feb 26, 2020 | Empty-Empty | N/A | 4 | No | N/A |  |
| Feb 27, 2020 | Empty-Empty | N/A | 4 | No | N/A |  |
| Feb 28, 2020 | Empty-Empty | N/A | 4 | No | N/A |  |
| Feb 29, 2020 | Empty-Empty | N/A | 4 | No | N/A | X |
| Mar 1, 2020 | Empty-Empty | N/A | 4 | No | N/A |  |
| Mar 2, 2020 | Empty-Empty | N/A | 4 | No | N/A |  |
| Mar 3, 2020 | Empty-Empty | N/A | 4 | No | N/A |  |

# **Table S2.** Simulated posterior means and 95% credible intervals for the estimated effect of feeder treatment as a function of temperature, AgeSex, days since the start of treatment on total daily feeder visits. The first treatment was comprised of two feeders filled with sunflower seeds at each of the eight feeder sites, and the second treatment was comprised of one full feeder at each of the eight sites. Daily foraging visits were taken as the sum of visits to all full feeders at the study site, per individual. Daily average temperature was measured in degrees Celsius. Estimated effects for each AgeSex category are presented in separate columns for ease of comparison, but values presented are derived from a single generalized linear mixed effect model with Poisson error distribution. Results from the same model fixed effect structure with Gaussian error distribution is presented in the main text (Table 1).

|  | **Adult  Male** | **Immature Male** | **Adult  Female** | **Immature Female** |
| --- | --- | --- | --- | --- |
| **Fixed effects** | **β (95% CrI)** | **β (95% CrI)** | **β (95% CrI)** | **β (95% CrI)** |
| Intercept^1^ | 4.72 (4.48, 4.97) | 4.86 (4.47, 5.34) | 4.91 (4.62, 5.11) | 4.86 (4.47, 5.34) |
| Feeders (1) | -0.46 (-0.59, -0.39) | -0.37 (-0.57, -0.09) | -0.47 (-0.59, -0.38) | -0.37 (-0.57, -0.09) |
| Feeders2:Temp^2^ | 0.00( -0.03, 0.04) | -0.07 (-0.14, 0.00) | 0.04 (-0.01, 0.07) | -0.07 (-0.14, 0.00) |
| Feeders1:Temp^2^ | 0.03 (-0.08, 0.10) | -0.01 (-0.25, 0.15) | 0.05 (-0.04, 0.14) | -0.01 (-0.25, 0.15) |
| Feeder2:Days^3^ | -0.10 (-0.15, -0.06) | -0.16 (-0.27, -0.05) | -0.08 (-0.13, -0.03) | -0.16 (-0.27, -0.05) |
| Feeders1:Days^3^ | -0.06 (-0.10, 0.00) | -0.06 (-0.17, 0.04) | -0.05 (-0.09, 0.01) | -0.06 (-0.17, 0.04) |
| **Random effects** | **σ (95% CrI)** | | | |
| ID | 0.51 (0.47, 0.57) | | | |
| Trial number | 0.02 (0.01, 0.03) | | | |
| row_id | 0.18 (0.17, 0.19) | | | |
| residual | 1 | | | |

1. Separate intercepts estimated for each age/sex category. Intercept estimates are for the two-feeder treatment, at the mean daily ambient temperature in the dataset (-11.53^o^C) and on the first day of the treatment.

2. Average daily temperature was measured in degrees Celsius and was centered and standardized prior to analysis. Thus, temperature effects are estimated for a change of 1 SD (6.72^o^C).

3. Days since start of treatment was left-zeroed prior to analysis so that the intercept was estimated on day 1. Estimated effect is change in daily feeder visits per day

# **Table S3.** Simulated posterior means and 95% credible intervals for the estimated effect of feeder treatment, AgeSex, and their interaction on total time spent foraging modelled with Gaussian errors. Separate intercepts (two-feeder treatment) and slopes (change from two-feeder treatment to single-feeder treatment) were estimated for each Age-Sex category during the two-feeder treatment to facilitate pairwise comparisons between all age-sex classes. Analyses in main text removed birds with low overall feeder visits to improve model fit. Analysis presented here includes all birds and is qualitatively similar to results presented in main text Table 2.

|  | **Foraging hours** |
| --- | --- |
| **Fixed effects** | **β (95% CrI)** |
| Adult-Male | 6.88 (6.22, 7.37) |
| Adult-Female | 7.02 (6.57, 7.71) |
| Immature-Male | 6.26 (6.29, 8.00) |
| Immature-Female | 7.21 (6.29, 8.16) |
| Feeders(1): |  |
| Adult-Male | 0.54 (0.37, 0.72) |
| Adult-Female | 0.44 (0.23, 0.58) |
| Immature-Male | 0.64 (0.20, 0.83) |
| Immature-Female | 0.93 (0.54, 1.34) |
| **Random effects** | **σ (95% CrI)** |
| ID | 1.66 (1.48, 1.85) |
| Trial | 0.16 (0.13, 0.24) |
| Residual | 1.87 (1.78, 1.99) |
|  |  |

# **Table S4.** Simulated posterior means and 95% credible intervals for the estimated effect of feeder treatment, AgeSex, and their interaction on total daily feeder visits modelled with Gaussian errors, for daily bird observations with a foraging window within +/- 2.5 hrs of daylength. Separate intercepts (two-feeder treatment) and slopes (change from two-feeder treatment to single-feeder treatment) were estimated for each AgeSex category during the two-feeder treatment to facilitate pairwise comparisons between all age-sex classes. Analyses in main text included all observations. Analysis presented here is qualitatively similar to results presented in main text Table 1.

| \|  \| **Adult  Male** \| **Immature Male** \| **Adult  Female** \| **Immature Female** \| \| --- \| --- \| --- \| --- \| --- \| \| **Fixed effects** \| **β (95% CrI)** \| **β (95% CrI)** \| **β (95% CrI)** \| **β (95% CrI)** \| \| Intercept^1^ \| 209 (191, 230) \| 261 (229, 291) \| 208 (190, 231) \| 193 (163, 230) \| \| Feeders (1) \| -121 (-132, -108) \| -147 (-169, -124) \| -104 (-119, -93) \| -91 (-113, -58) \| \| Feeders2:Temp^2^ \| 11 (6, 15) \| 5 (-3, 14) \| 7 (3, 11) \| 13 (5, 23) \| \| Feeders1:Temp^2^ \| 3 (-5, 13) \| 13 (-2, 35) \| 7 (-2, 18) \| 0 (-25, 19) \| \| Feeder2:Days^3^ \| -46 (-53, -40) \| -48 (-57, -34) \| -34 (-40, -28) \| -31 (-43, -15) \| \| Feeders1:Days^3^ \| -5 ( -11, 1) \| -7 (-18, 3) \| -8 (-13, -1) \| -5 (-19, 7) \| \| **Random effects** \| **σ (95% CrI)** \| \| \| \| \| ID \| 2016 (1726, 2216) \| \| \| \| \| Trial number \| 173 (119, 255) \| \| \| \| \| Residual \| 2396 (2276, 5559) \| \| \| \| |  |
| --- | --- | --- | --- | --- | --- | --- | --- | --- | --- | --- | --- | --- | --- | --- | --- | --- | --- | --- | --- | --- | --- | --- | --- | --- | --- | --- | --- | --- | --- | --- | --- | --- | --- | --- | --- | --- | --- | --- | --- | --- | --- | --- | --- | --- | --- | --- | --- | --- | --- | --- | --- | --- | --- | --- | --- | --- | --- | --- | --- | --- | --- |

1. Separate intercepts estimated for each age-sex category. Intercept estimates are for the two-feeder treatment, at the mean daily ambient temperature in the dataset (-11.53^o^C) and on the first day of the treatment.

2. Average daily temperature was measured in degrees Celsius and was centered and standardized prior to analysis. Thus, temperature effects are estimated for a change of 1 SD (6.72^o^C).

3. Days since start of treatment was left-zeroed prior to analysis so that the intercept was estimated on day 1. Estimated effect is change in daily feeder visits per day


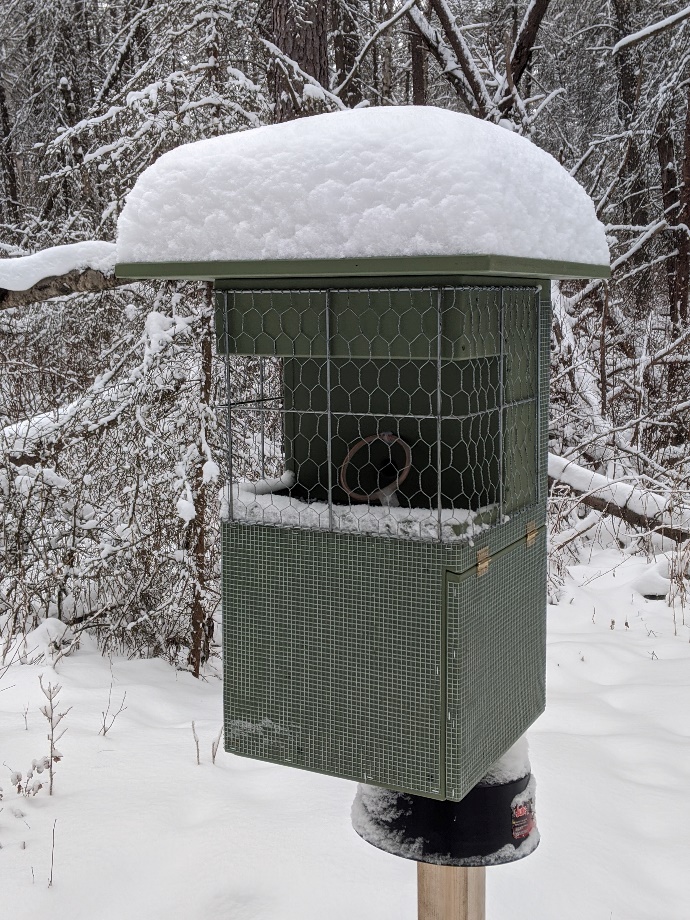


# **Figure S1**: Photo showing feeders used in the study. The copper circle is the RFID antenna, which provides a perch for birds to sit on while accessing sunflower seeds from a small opening behind it (3 cm diameter). The hinges visible on the right lower side of the feeder open a compartment where the RFID board with SD card and battery are located. The back and top of the feeder provide a large reservoir of sunflower seeds such that the feeders do not become depleted between successive observer visits (every 4 days). Feeders are protected in wire mesh to prevent destruction by squirrels. The chicken wire is used to restrict access to the area where sunflower seeds are available by non-focal species, but allows chickadees to pass through freely.

**
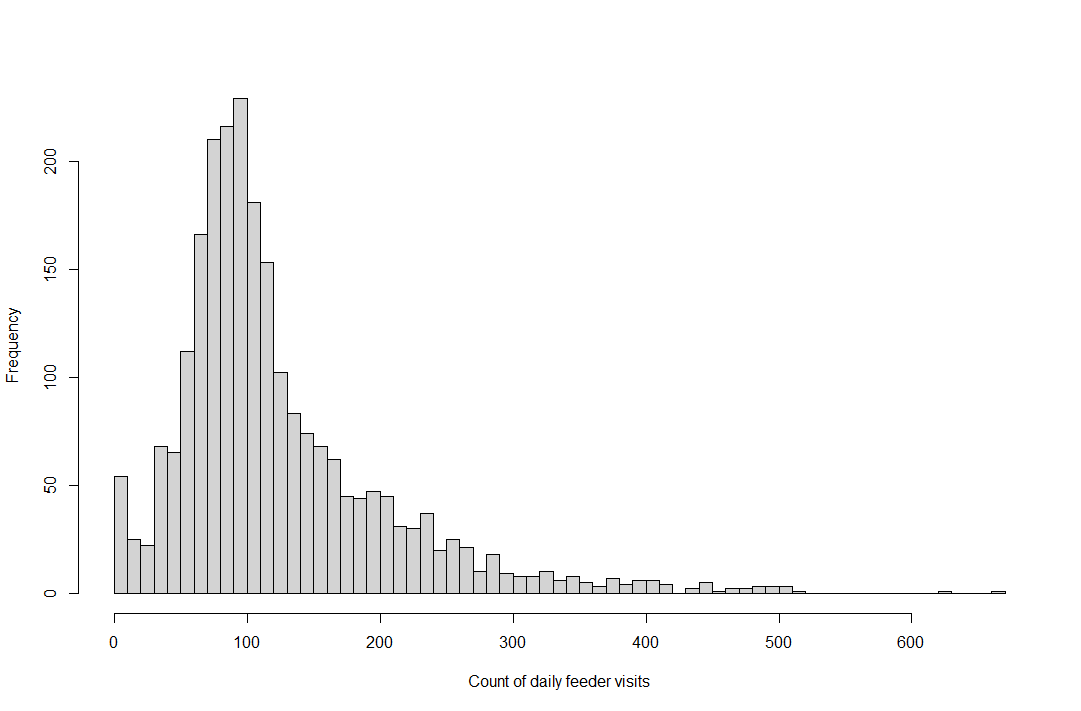
**

# Figure S2: Histogram of the count of daily feeder visits per bird.


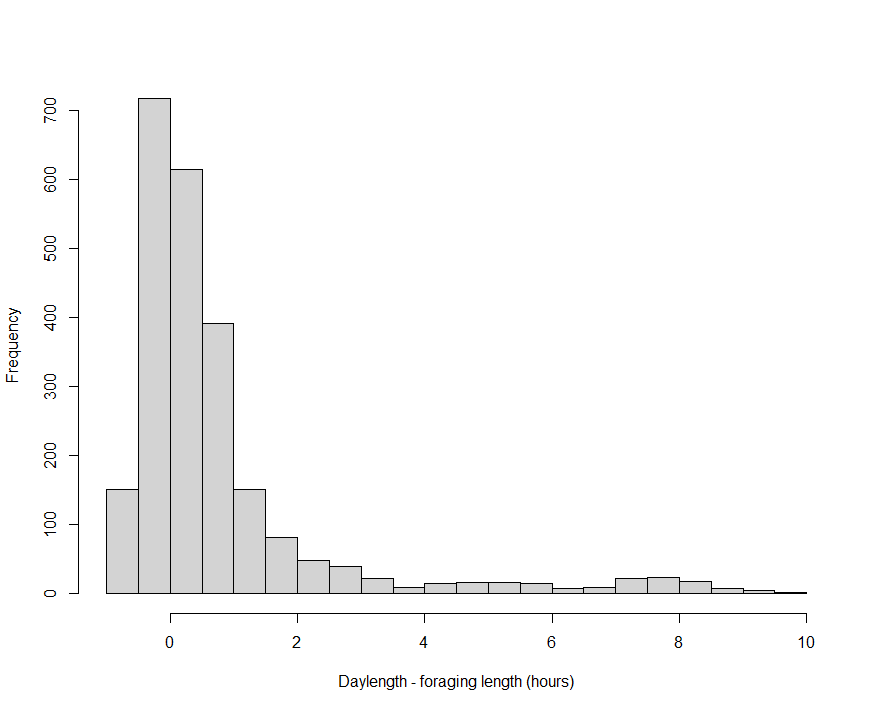


# Figure S3: Histogram of difference between total daylength (hours from sunrise to sunset) and foraging hours (hours between first and last feeder detection per bird per day). Most birds have foraging lengths within +/- 2.5 hours of daylength, with a long tail of longer differences. These birds had very short foraging windows at feeders, suggesting they were not heavily reliant on feeders.


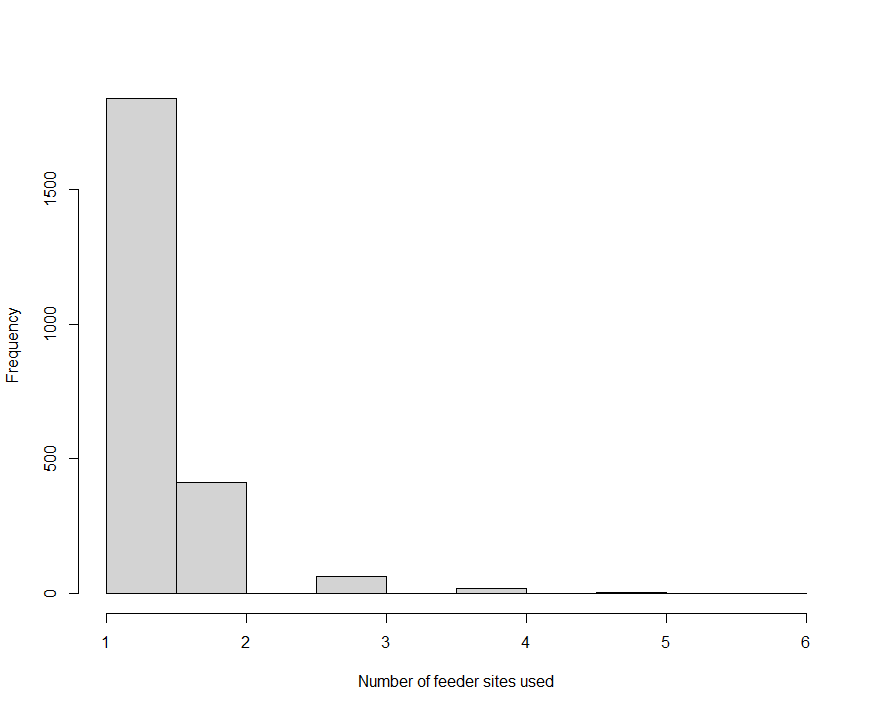


# Figure S4: Histogram of the count of feeder sites used per bird per day. Values were heavily skewed towards 1, therefore, data were modelled as binary (1 site used or >1 site used).


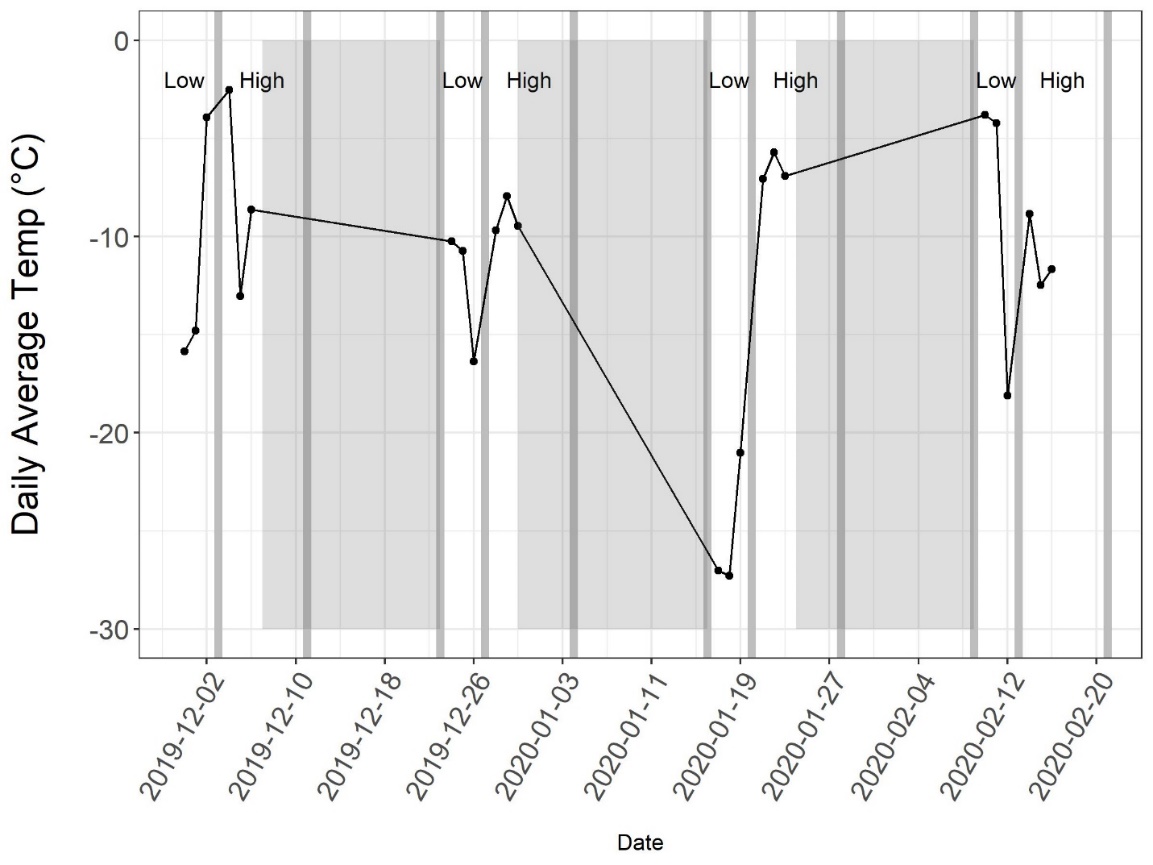


# **Figure S5.** Daily average temperature over the course of the competition experiment. Low (two feeders per site) and high (one feeder per site) competition treatment days are labelled, and grey shading indicates periods at which time data were not collected for this study. By chance, there was a significant temperature drop during the third round of the low competition treatment, driving a significant difference in average temperatures between low and high competition conditions.
